# Advances in Anti-Wrinkle Finishing Agent for Natural Fabrics

**DOI:** 10.3390/polym18030407

**Published:** 2026-02-04

**Authors:** Haoqian Luo, Haifeng Sun, Man Zhang, Jiating Wen, Mengmeng Chen, Jian Fang, Zhe Sun

**Affiliations:** 1 College of Textile and Clothing Engineering, Soochow University, Suzhou 215123, China; 2 National Local Joint Engineering Laboratory for Advanced Textile Processing and Clean Production, Wuhan Textile University, Wuhan 430200, China; 3 Glorious Sun Guangdong School of Fashion, Huizhou University, Huizhou 516007, China; 4 Transfar Zhilian Co., Ltd., Hangzhou 311215, China

**Keywords:** natural fabrics, anti-wrinkle, balanced performance, environmentally friendly process, multi-functional integration

## Abstract

Natural fabrics such as cotton and silk have been widely used due to their excellent properties, but their tendency to wrinkle limits their value. Traditional anti-wrinkle finishing agents suffer from issues like formaldehyde release and performance imbalance. This paper reviews the advances in anti-wrinkle finishing of cotton and silk fabrics, analyzing from the perspectives of environmentally friendly finishing agents, physical properties balancing, sustainable anti-wrinkle finishing, and synchronized multi-functionality. Current research have developed various environmentally friendly formaldehyde-free finishing agents, such as carboxylated polyaldehyde sucrose and α-lipoic acid, through strategies including natural product modification and organic–inorganic hybridization. The application of these agents can enable fabrics to achieve a balance between wrinkle resistance, mechanical properties, hydrophilicity, and resistance to yellowing properties. Simultaneously, they also overcome the limitations of traditional processes, endow fabric with integrated application of wrinkle resistance alongside functions such as dyeing, flame retardancy, and antibacterial properties. Moreover, optimization methods such as response surface methodology (RSM) have facilitated the precise regulation of process parameters. Future research should continue to focus on greenization, high performance, and multi-functional coordination, deepen molecular design and process optimization, and provide support for the sustainable development of the textile industry.

## 1. Introduction

Textiles permeate all aspects of human life, including clothing, food, housing, and transportation, serving as a crucial component of the national economy [1,2]. Among them, cotton fabrics have long maintained a core position in the global textile and apparel market due to their excellent moisture absorption and breathability, soft wearing comfort, and biodegradability. Meanwhile, silk fibers and their fabrics, endowed with unique pearlescent luster, superior wear comfort, good biocompatibility, and natural origin, combine practical value with high-end positioning. They are long regarded as high-grade textile products and widely applied in luxury apparel, high-end home textiles, and ceremonial articles [3,4,5]. However, despite their distinctive advantages and important positions in the textile field, both cotton and silk fabrics share a critical common shortcoming; they are both prone to wrinkling, which has become a key issue restricting their application experience and product added-value [6]. For cotton, cellulose molecular chains are connected by strong hydrogen bonds, with the amorphous region accounting for about 30–40%. Hydrogen bonds are prone to breakage under external forces, causing molecular chain slippage, and it is difficult to re-form a stable hydrogen bond network after the removal of external forces. The elastic recovery rate of cotton fibers is only about 70%, failing to rebound quickly after deformation. Additionally, cotton fibers exhibit high hygroscopicity; the amorphous region swells upon moisture absorption, further weakening hydrogen bond interactions and making irreversible wrinkles more likely to occur, ultimately leading to obvious and non-recoverable wrinkles in cotton fabrics [7]. For silk fabrics, silk fibroin has low component crystallinity with numerous internal voids and contains abundant salt bonds and hydrogen bonds in the amorphous region without chemical cross-linking. Under the action of water molecules or external forces, these salt bonds and hydrogen bonds are disrupted, resulting in relative slippage between polymer chains. This slippage causes polar groups between amino acids to reattach at new positions, so when external forces disappear, the resulting deformation cannot recover to its original shape, thus forming wrinkles [8].

To address this problem, anti-wrinkle finishing has become a key process in the post-finishing of cotton and silk fabrics. Nevertheless, traditional anti-wrinkle finishing technologies face multiple bottlenecks: for instance, formaldehyde-containing finishing agents (e.g., dimethyloldihydroxyethylene urea (DMDHEU)) achieve significant anti-wrinkle effects but pose health risks due to formaldehyde release; formaldehyde-free alternatives (e.g., citric acid (CA), 1,2,3,4-butanetetracarboxylic acid (BTCA)) suffer from high costs, severe fabric strength loss, yellowing, or insufficient wash fastness [9]. More importantly, as wearable fabrics, they are required to balance compatibility with dyeing, printing, and other processes, as well as application properties such as flame retardancy, moisture absorption, breathability, and antibacterial activity while achieving anti-wrinkle performance, which presents great challenges.

In recent years, with the deepening of the concepts of eco-friendly, high-value-added, and functional textiles and the upgrading of consumer demands, the development of eco-friendly formaldehyde-free anti-wrinkle finishing technologies with balanced multi-performance, integrated functionality, and high process efficiency has become a research hotspot [10]. Researchers have conducted systematic explorations around three main directions: molecular design of finishing agents, innovation of cross-linking mechanisms, and collaborative optimization of processes. Strategies such as natural product modification, organic–inorganic hybridization, and multi-functional integrated finishing have provided new approaches to break through the limitations of traditional technologies [11].

Based on research achievements in the field of cotton fabric anti-wrinkle finishing in recent years, this paper systematically reviews the research progress from the dimensions of environmentally friendly finishing agents, physical properties balancing, sustainable anti-wrinkle finishing, and synchronized multi-functionality (Table 1). Furthermore, it analyzes current technical bottlenecks and predicts future development trends. It aims to provide references for the research, development, and industrial application of high-performance, eco-friendly anti-wrinkle finishing technologies for cotton fabrics.

## 2. Advances in Textiles Anti-Wrinkle Finishing

### 2.1. Anti-Wrinkle Combined with Physical Properties

Traditional anti-wrinkle finishing agents, such as DMDHEU, tend to release formaldehyde, posing potential safety risks. On the other hand, fabric anti-wrinkle finishing is typically a cross-linking process. With the increase in cross-linking degree, both the number of functional groups on fiber macromolecules surface and the structure of crystalline regions are altered, which will also affect the physical properties of fabrics, such as mechanical strength and hydrophilicity [23]. Therefore, the development of eco-friendly anti-wrinkle finishing agents together with the balance of performance are of vital importance.

#### 2.1.1. Wrinkle Resistance Combined with Mechanical Property

Regarding mechanical properties, on one hand, after anti-wrinkle finishing, the reactive groups on fiber have been covalently cross-linked. These cross-linking points will restrict the sliding and elastic deformation of molecular chains. This further weakens the fiber’s ability to disperse stress through molecular chain movement under external forces, ultimately manifesting as fiber embrittlement and diminished mechanical performance [24]. On the other hand, some traditional cross-linking agents (e.g., CA, BTCA) require reaction under acidic conditions. Such an environment is prone to catalyze the hydrolysis of cellulose glycosidic bonds, leading to a decrease in the degree of polymerization of the fiber and a reduction in mechanical strength. In addition, as for finishing processes, curing at a high temperature may accelerate an oxidative degradation of cellulose and intensify stress concentration, while inappropriate padding pressure can bring physical damage to fibers, further weakening fabric strength [25,26]. Based on the above, attention has been paid to the molecular design of finishing agents, material composites, and processes.

In molecular design, by using sucrose as raw material, Lou et al. [12] prepared a novel carboxylated polyaldehyde sucrose (openSu) finishing agent containing multiple aldehyde and carboxyl groups via two-step oxidation catalyzed by 2,2,6,6-tetramethyl-1-piperidinyloxy (TEMPO)-laccase and sodium periodate (NaIO_4_). In this meticulously designed molecule, aldehyde groups underwent etherification with hydroxyl groups of cotton fibers, while carboxyl groups undertook esterification with hydroxyl groups. These two cross-linking approaches synergistically endowed fabric with a flexible cross-linked network instead of a single rigid cross-linkage. Benefiting from this, the treated fabric exhibited a crease recovery angle (CRA) of 258° (comparable to that of DMDHEU and glyoxal (GA)), a whiteness index (WI) of 72.1, a tensile strength retention (TSR) rate > 65%, a wetting time < 3 s, and a stiffness of 5.65 cm. Zhang et al. [13] prepared a kind of isocyanate-terminated polysiloxane (ITPSI) by integrating flexible hydrogen-terminated polysiloxane (HTPSI), methylallyl alcohol, and diphenylmethane diisocyanate (MDI). Under optimized conditions, the dry crease recovery angle (DCRA)/wet crease recovery angle (WCRA) of the fabric with flexible cross-linking reached 302.6° and 229°, which were 82.6° and 64° higher than those of untreated fabric, respectively. It is worth noting that the strength retention rate of treated fabric was as high as 99.0%, indicating that this is an almost non-destructive anti-wrinkle method. Further, Liu et al. [14,15] synthesized three polycarboxyl-terminated trichlorotriazine derivatives (Compounds 1 (one-substitution), 2 (di-substitution), and 3 (tri-substituted)) with dual active sites of chlorine atoms and carboxyl groups, using cyanuric chloride and iminodiacetic acid as raw materials. A formaldehyde-free and eco-friendly anti-wrinkle finishing system for silk fabric was also developed. It was found that Compound 3 exhibited the optimal performance when cured at 165 °C for 3 min: the WCRA reached 211.3° (significantly higher than the 169.7° of BTCA group), and the DCRA was 328.3° (comparable to the 328.7° of BTCA group). The maximum TSR rate of fabrics finished with the three derivatives was 98.3% (superior to 88.0% of the BTCA group), and the WI ranged from 79.2 to 80.2 (basically consistent with 80.2 of untreated fabrics, with no yellowing). After 10 wash cycles, the DCRA/WCRA of the fabric treated by Compound 3 were 343.6° and 187.9°, respectively. After 20 wash cycles, these values remained at 343.3° and 167.6°, demonstrating superior wash resistance compared to the BTCA group.

At the same time, material composites can often overcome the limitations of individual materials. Peng et al. [16] developed a composite finishing system consisting of maleic acid (MA) and NaH_2_PO_2_. Flexible cross-linking was achieved through a two-step reaction: Step I (low-temperature esterification): MA underwent esterification with hydroxyl groups of cotton fibers at a relatively low temperature 100~140 °C, forming single ester bond linkages; Step II (high-temperature cross-linking): When the temperature rised above 140 °C, the H-P- residues of NaH_2_PO_2_ underwent an addition reaction with the C=C in MA, combining two esterified MA molecules to form flexible P-C cross-linking bonds. Under this strategy, the finished plain weave fabric exhibited a CRA of 275° (comparable to 274° for DMDHEU-treated fabric), warp TSR rate of 52%, weft TSR rate of 46%, and tear strength retention rate of 57%. Both of them were higher than that of the DMDHEU-treated group. Islam et al. [17] constructed a hybrid finishing system composed of etherified DMDHEU resin, waterborne polyurethane (WPU), and maleic anhydride-grafted polyethylene wax (PEW-g-MAH, for lubrication). Functional modification of cotton fabrics was achieved via a one-step padding-drying-curing process. Through parameter optimization, the optimal formulation was determined as follows: 100 g/L DMDHEU + 40 g/L WPU + 15 g/L PEW, cured at 160 °C for 3 min. Under these conditions, the wrinkle recovery rate was 83.71%, higher than that of untreated fabric (78.27%) and the DMDHEU/WPU composite system (77.27%). The tensile strength loss was only 18%, far lower than 41% of the fabric treated by DMDHEU alone. Chakraborty et al. [18] adopted the Box–Behnken experimental design combined with RSM to comparatively investigate the anti-wrinkle effects of two cross-linking agents (modified DMDHEU and CA) and optimized the process parameters to balance durable press (DP) and physical properties. Modified DMDHEU formed crosslinks with cellulose through etherification reaction, while CA formed crosslinks with cellulose through an esterification reaction; both DMDHEU and CA systems utilized catalysts, silicone softeners (SS), and polyethylene emulsion (PE). Under optimal conditions, the modified DMDHEU-finished fabric achieved a DP rating of 3.7, a total CRA of 263°, a warp TSR rate of 77.03%, and a weft TSR rate of 84.87%. While for CA treated samples, the DP rating was 3.7, the CRA was 223°, and the warp TSR rate was 74.92%. SS and PE showed no significant effect on the DP rating but could improve the fabric’s hand feel and strength.

In addition to materials, the regulation and design process and introduction of catalysts/additives are equally important. Ibrahim et al. [27] used CA as cross-linking agent to investigate the effects of catalytic systems, additives, cross-linking agent ratios, curing conditions, and substrate types on the DP performance of cellulosic fabrics. Results showed that when the CA concentration was 80 g/L and curing was conducted at 180 °C for 90 s, the cotton fabric exhibited a carboxyl content of 82.0 mEq/100 g, a DCRA/WCRA of 242°/225°, a DP rating of 3.5, a tear strength retention rate of 68.5%, and a WI of 58.6%. After adding 15 g/L triethanolamine hydrochloride (TEA·HCl, which reduced the content of free carboxyl groups), the DCRA/WCRA could increase to 260°/244°. The addition of 30 g/L polyethylene glycol 600 (PEG-600, whose hydroxyl groups competed with CA carboxyl groups for esterification, forming flexible cross-linking bridges and reducing stress concentration) resulted in a CRA of 262° and a tear strength retention rate of 72.4%. The silicone softener could further improve the strength retention rate to 82.5%. Eyupoglu et al. [28] developed a composite anti-wrinkle finishing system combining oxygen plasma pretreatment and formaldehyde-free resin. The fabric performance was improved after oxygen plasma pretreatment at 100 W for 1–5 min followed by finishing with three different resins. Optimal combination was determined as 1 min plasma pretreatment + resin Formulation A (120 g/L PROTOREZ FFO 01 + 24 g/L CURITE 5184, etc.), under which the warp CRA and the weft CRA reached 113.5 ± 3.5° and 102 ± 3°, respectively, showing a significant increase compared with the non-plasma-treated group. To address the issues of insufficient cross-linking efficiency and excessive costs during BTCA wrinkle-resistant finishing of cotton fabrics, Xiao et al. [29] employed NaH_2_PO_2_ as a catalyst and introduced neutral salts NaCl and Na_2_SO_4_ as additives to promote the cross-linking reaction between BTCA and cellulose, so as to enhance wrinkle resistance. Based on the Donnan equilibrium model, neutral salts could neutralize the negative charges on fiber surface, thus reducing the repulsive effect of BTCA anions. This promoted their adsorption and diffusion into the fiber interior, weakened the intermolecular hydrogen bonds of BTCA, and catalyzed the formation of five-membered cyclic anhydrides (active intermediates), thereby enhancing ester bond cross-linking. After such improvements, the CRA of treated fabric reached 251 and 248°, respectively, which was approximately 12° higher than that of the additive-free system. Ke et al. [30] employed GA as a cross-linking agent and MgCl_2_ as a catalyst to impart wrinkle resistance to Tencel fabric. Box–Behnken experimental design and RSM was adopted to optimize the finishing scheme. Under optimized conditions, the CRA of Tencel fabric could reach 137.4°, representing a 47.1% increase compared with the untreated sample (93.4°).

In addition, Tusief et al. [31] systematically investigated the influence of finishing agent type, application process, and concentration on the warp/weft tear strength of fabrics. Four modified N-methyldihydroxyethylene urea-based commercial finishing agents (Texicil DC, Knittex RCT, Arkofix NEC, Arkofix ELF) and three processes (Pad-dry, Pad-dry-cure, Pad-flash-cure), together with a concentration range of 60–120 g/L, were designed. Results indicated that Arkofix ELF demonstrated optimal performance, showing an average warp/weft tear strength of 8.1251 N/7.3186 N. The Pad-flash-cure process provided the best protective effect, yielding corresponding average values of 8.0556 N and 7.0607 N. Low concentrations (60 g/L) could reduce mechanical damage, bring tear strengths of 8.1760 N/7.2589 N, while concentrations at 120 g/L decreased these values to 7.8562 N/6.6831 N. The optimal combination was Arkofix ELF (60 g/L) + Pad-flash-cure process; in this condition, tear strengths of 8.1251 N/7.3186 N in both warp and weft directions could be achieved. This strategy maximized mechanical properties while delivering wrinkle resistance. Xu Lei et al. [32] developed a composite polycarboxylic acid anti-wrinkle finishing agent Y with a molar ratio of CA-to-MA of 1:2, which effectively balanced anti-wrinkle properties, mechanical performance, and durability. Under MgCl_2_ catalysis, ester bonds were formed between the carboxyl groups on Y and the amino/hydroxyl groups on silk fibers, thereby restricting molecular chain slip. The DCRA and WCRA of treated silk reached 270° and 253°, respectively (representing increases of 33% and 37.5% compared with untreated silk). In addition, this finishing agent exhibited excellent wash resistance: after 50 washing cycles, the DCRA and WCRA still remained at 261° and 242°.

#### 2.1.2. Wrinkle Resistance Combined with Hydrophilicity

After wrinkle-resistant finishing, the number of hydroxyl groups in fibers decreases due to cross-linking. This reduction will diminish the fabric’s moisture absorption capacity and then affect its wear comfort.

In response to this, Shih et al. [33] applied hydrophilic PEG to the wrinkle-resistant finishing of fabrics, and the effects of PEG molecular weight (400, 600, 1000, 1500), concentration (0–30%), curing temperature (180–210 °C), and curing time (10–30 s) on fabric hygroscopicity, DCRA, WCRA, and TSR were studied. Results showed that, after adding PEG, hygroscopicity was improved by more than 20% compared with the traditional finishing method. Within the range of low molecular weight (400–1000) and low concentration (≤10%), hygroscopicity and anti-wrinkle properties increased with the rise in PEG molecular weight and concentration. Increasing the curing temperature could enhance hygroscopicity and anti-wrinkle properties but reduce strength retention. The optimal process was determined as PEG 1000 (10%) with curing at 200 °C for 30 s, under which the fabric exhibited a liquid absorption length of 7.8 cm, a DCRA of 290°, a WCRA of 274°, and a TSR of 60.6%. Further, Huang et al. [34] proposed an innovative strategy using α-lipoic acid (ALA) as a formaldehyde-free cross-linking agent for the first time. ALA was grafted onto the surface and inside of cotton fibers via esterification between its carboxyl groups and the hydroxyl groups of cotton fibers; disulfide bonds were cleaved to generate thiol groups by reduction with sodium borohydride (NaBH_4_), which were then reconstructed into topological disulfide bonds and a small amount of hydrophilic sulfonic acid groups through air oxidation, forming a composite structure of an internal cross-linked network and a surface-anchored coating. The effects of ALA concentration and NaBH_4_ dosage were systematically investigated. Under optimal conditions (0.75 mol/L ALA), the CRA reached 252.29°, nearly a 100% increase compared with the untreated fabric 126.55°. In terms of durability, the CRA remained at 240.53° after 20 washing cycles; the TSR exceeded 100% in all cases, with a maximum of 108.3%, which was far superior to the BTCA (42.4%) and DMDHEU (46.8%) systems. In addition, the wetting time was only 4.25 s, exhibiting excellent hydrophilicity.

#### 2.1.3. Wrinkle Resistance Combined with Anti-Yellowing

When CA is used as a formaldehyde-free cross-linking agent for cotton fabric treatment, the α-hydroxyl groups in its molecules tend to suffer dehydration during high-temperature curing at 140–180 °C, generating unsaturated aconitic acid (AA) containing conjugated double bonds [35]. This conjugated structure alters the fabric’s light absorption properties in visible spectrum, resulting in significant yellowing of cotton fabric, and the degree of yellowing intensifies with increasing curing temperature and prolonged treatment time. Meanwhile, unreacted residual CA may further decompose or polymerize at high temperatures, aggravating the yellowing phenomenon. This problem has become a bottleneck, restricting the large-scale application of CA in the wrinkle-resistant finishing of cotton fabrics.

In view of this, Ahmed et al. [36] constructed a composite finishing system of CA and silk fibroin solution. Silk fibroin molecules contained a large number of active groups such as amino and hydroxyl groups; under certain conditions, these groups preferentially underwent cross-linking reactions with the carboxyl groups of CA (forming ester or amide bonds) and then reduced the probability of intramolecular dehydration of CA itself to form unsaturated acids. Under this strategy, the DCRA of treated fabric could reach 252°, together with a warp TSR rate of 84%, weft TSR rate of 82%, warp tear strength retention rate of 96%, and weft tear strength retention rate of 93%. The WI was 75, retaining 93% whiteness of the untreated fabric. An over 25% whiteness loss caused by CA finishing alone was effectively solved. Zhang et al. [37] synthesized two formaldehyde-free CA dimer finishing agents (CACA-HMDI, CACA-IPDI) by reacting isocyanates with yellowing-prone “hydroxyl groups” in CA molecules. Using NaH_2_PO_2_ as catalyst, a padding-pre-drying-curing process was adopted to optimize the finishing process. Under optimized conditions, the CRA of finished fabric reached 275° and 251°, respectively, which were significantly higher than 240° of the CA-alone treatment. Meanwhile, WI values reached 71 and 70; yellowing was effectively suppressed. After seven washing cycles, the CRA retention rates were 93.45% (257°) and 88.84% (223°), demonstrating good washing resistance. Yao et al. [38] proposed an improved scheme by using polyols as chain extenders. They attempted to inhibit the formation of unsaturated bonds by reacting hydroxyl groups with CA to form ester bonds. Results showed that the fabric treated with pure CA showed a CRA of 257°, a WI of 38.6, and a DP rating of 3.3. After adding xylitol, the CRA increased to 270°, the WI reached 57.8, and the DP rating rose to 3.9, which was close to the BTCA (CRA 277°, WI 62.8) and DMDHEU (CRA 275°, WI 64.2). Subsequently, Liu et al. [39] also constructed a green cross-linking system composed of CA and xylitol. By optimizing process parameters through RSM, they achieved pilot-scale application. In the verification test, the CRA reached 269.8°, the WI was 58.5, and the strength retention was 6.19 N, with deviations from the predicted values ≤ 3.9%. Pilot-scale results showed that, after finishing, bleached jacquard cotton fabric exhibited a CRA of 292°, a DP rating of 3.5, and dimensional stability of −0.90%, which were comparable to the effect of commercially available low-formaldehyde resin F-ECO; the tear strength was 10.0 N and the breaking strength was 290 N, significantly superior to F-ECO (7.5 N, 180 N), with a WI of 75.4 and no obvious yellowing. Tang et al. [40] also adopted alkaline hydrogen peroxide (H_2_O_2_) bleaching technology to improve whiteness while balancing anti-wrinkle properties and mechanical performance. They investigated the regulation of bleaching system parameters including pH value (8.0–12.0), temperature (60–98 °C), and time (10–50 min), and revealed the dual-action mechanism. Results indicated that after CA treatment, the fabric’s CRA increased from 202.6° to 287.3°, while the WI value decreased from 89.23 to 68.86 and the b* value rose from 1.28 to 6.24. Under pH = 12.0 conditions, after H_2_O_2_ bleaching treatment, the WI value reached 85.12, the b* value decreased to 1.99, and the CRA decreased to 236.3°; at a bleaching temperature of 98 °C and a bleaching time of 10 min, the WI was 80.8 and the b* value was 3.21. Extending the treatment time had no significant effect on whiteness, but the CRA decreased. The optimal process was determined as pH = 10.5, 98 °C, and 10 min, under which the WI reached 81.84; the CRA was maintained at 244.8°, and there was no obvious loss in strength retention rate, achieving a good balance between whiteness and anti-wrinkle properties.

To further address potential risks such as cross-link bond breakage and anti-wrinkle property degradation that may arise from high-temperature H_2_O_2_ bleaching, Luo et al. [41] developed a low-temperature post-bleaching system using peroxide activated by N-[4-(triethylammoniomethyl) benzoyl] caprolactam chloride (TBCC), and proposed a “one-step post-bleaching” process to brighten yellowed fabrics and retain anti-wrinkle properties under mild conditions. A low-temperature post-bleaching system of TBCC/H_2_O_2_/NaHCO_3_ was constructed (30 mmol/L TBCC, 33 mmol/L H_2_O_2_, 36 mmol/L NaHCO_3_), with process conditions of 50 °C, 40 min, bath ratio 20:1, and addition of penetrant JFC and stabilizer DM-1403. After “one-step post-bleaching”, finished fabrics achieved a whiteness of 75.10, CRA of 221°, strength retention rate of 60%, wettability of <1 s, and DP rating of 2.5. These properties were comparable to those of the “two-step bleaching” process (whiteness 82.16, CRA 222°, TSR 62%), while removing the pre-bleaching step, thus saving energy and time. In addition, Wang et al. [42] proposed a mixed polycarboxylic acid finishing system of BTCA/CA, revealing the mechanism of CA action through kinetic analysis, performance testing, and characterization. Results showed that within the temperature range of 120–180 °C, the activation energy of esterification reaction for the mixed system was 40.7 kJ/mol, lower than that of the single BTCA system (44.4 kJ/mol), indicating that the reaction of the mixed system was easier to proceed. The optimal formulation was 50 g/L BTCA + 45 g/L CA + 38 g/L NaH_2_PO_2_; when cured at 170 °C for 90 s, the CRA was significantly improved, and the fabric strength slightly increased at a CA concentration of 40 g/L, reflecting the chain extender effect of CA. The yellowness index of fabrics treated with the mixed system was 11.53, lower than that of single CA treatment (13.26).

### 2.2. Environmental-Friendly Anti-Wrinkle Finishing

**Figure 1:**
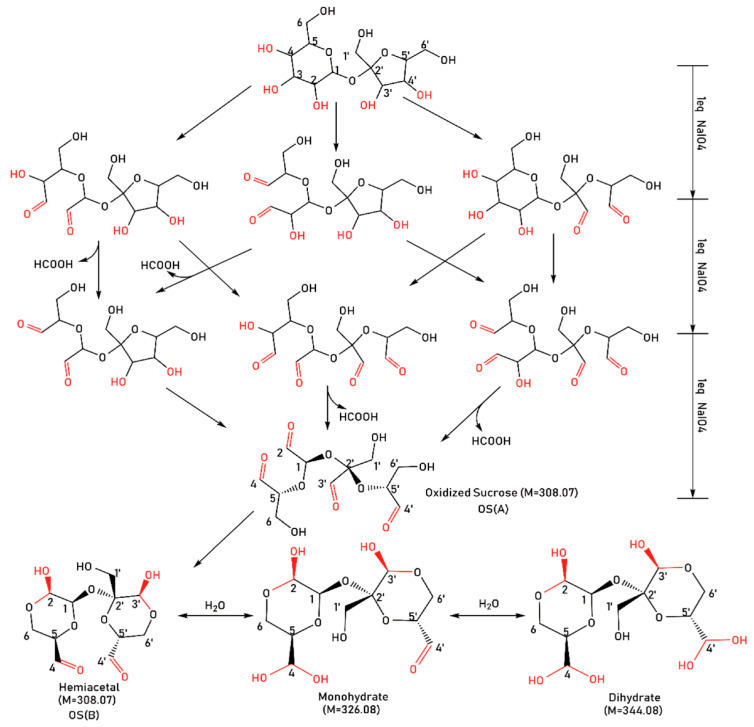
Reaction pathways for oxidized sucrose preparation [20] (Mao Feng et al., Polymers, 2022, 14(14): 2842).

With the continuous advancement of eco-friendly dyeing and finishing, attention has also been paid to the sustainability of fabric anti-wrinkle finishing agents and the clean finishing process. Xu Lei et al. [43] addressed the inherent tendency of silk to wrinkle by utilizing renewable and skin-friendly sericin as an eco-friendly anti-wrinkle finishing agent, accompanied with triethanolamine (TEA) as an adjuvant. Sericin formed covalent cross-linking with active groups in silk fibers through its hydroxyl and carboxyl groups, restricting the sliding of fiber molecular chains and thereby enhancing wrinkle resistance. The DCRA of treated silk reached 256° and the WCRA reached 222° (representing increases of 38.4% and 44.2%, respectively, compared to untreated silk). Boondaeng et al. [19] used empty palm oil fruit bunches (EPOFB)—an agricultural waste as raw material to biosynthesize itaconic acid (IA) via Aspergillus terreus K17. An eco-friendly anti-wrinkle finishing agent for cotton fabrics was successfully developed. The bio-synthesized IA could form ester bonds with the hydroxyl groups of cotton fibers via anhydride intermediates, thus restricting molecular chain slippage and enhancing wrinkle resistance. The optimal process was determined as follows: 8% *w*/*v* biosynthesized IA + 8% *w*/*v* NaH_2_PO_2_, two-dip–two-nip padding (pick-up rate 90%), drying at 85 °C for 3 min followed by curing at 180 °C for 2 min. Under these conditions, the CRA of treated fabric reached 158.2° (showing a 45.4% increase compared with untreated fabric at 108.83°). Although the anti-wrinkle effect of biosynthesized IA is slightly inferior to that of BTCA and MA, it realizes the resource utilization of agricultural waste, providing a sustainable path for green anti-wrinkle finishing of cotton fabrics. Feng et al. [20] used renewable and non-toxic sucrose as raw material to synthesize tetra-aldehyde oxidized sucrose (OS) via selective oxidation with sodium periodate, developing a formaldehyde-free and eco-friendly anti-wrinkle finishing agent (Figure 1). Sucrose oxide existed in the form of hydrate and could undergo isomerization to form a dihexane hemiacetal ring structure. This structure cross-linked with the hydroxyl groups of cotton fibers, restricting molecular chain slippage and ultimately achieving wrinkle resistance. The CRA of finished cotton fabric reached 260° (showing a 96.9% increase compared with untreated fabric at 132°).

Considering the issue that BTCA anti-wrinkle finishing agent needs to be used in conjunction with phosphorus-containing catalyst. Schramm et al. [21] developed a formaldehyde-free organic–inorganic hybrid anti-wrinkle finishing agent composed of BTCA and 3-aminopropyltriethoxysilane (APTES), which did not need additional catalysts. BTCA and APTES formed polyacrylamide (PAA) through a sol–gel reaction. During high-temperature curing, imide groups and five-membered cyclic anhydride intermediates were generated and then cross-linked with the hydroxyl groups of cotton fibers to form ester bonds, thereby achieving wrinkle resistance. Under optimized conditions, the DCRA reached 243.6° (27.7% increase compared with untreated fabric at 190.8°), the TSR rate was 78.5%, and the WI was 17.1. Cai et al. [22] synthesized a silicon-containing epoxy finishing agent (EPSIB), and developed an aqueous, eco-friendly anti-wrinkle finishing scheme that balanced anti-wrinkle properties, hand feel, and durability. The EPSIB cross-linked with amino and hydroxyl groups of silk fibers via epoxy groups, and its siloxane segments could optimize the fabric hand feel without using any organic solvents. The DCRA of the finished silk reached 267°, and the WCRA value reached 275°, representing increases of 21.4% and 44.7%, respectively, compared to untreated silk.

### 2.3. Anti-Wrinkle Combined with Other Dyeing and Finishing Process

The decline in dyeing performance of textiles after anti-wrinkle finishing has long been a technical pain point in the industry, and its core cause stems from the interference of the interaction mechanism between anti-wrinkle finishing agents and fibers with the dyeing system. Li et al. [44] proposed a scheme that used 2,4,6-trichloropyrimidine (TLP) as a formaldehyde-free anti-wrinkle finishing agent to reduce the occupation of dyeing sites and balanced anti-wrinkle properties with dyeing performance. TLP emulsion (average particle size 287.9 nm, polydispersity index 0.183) was prepared via ultrasonic dispersion and finishing process parameters were optimized. Results showed that, after finishing, the fabric exhibited a DCRA of 268.1° and a WCRA of 247.2°, representing an increase of 16–20% compared with untreated fabric (223.4°, 198.5°), and the CRA could remain at 241.6° and 220.3° after 20 washing cycles. When adopting the “finishing–dyeing” process, the K/S value reached 14.08 (11.38 for only-dyeing-fabric), the maximum exhaustion rate of reactive dyes was 91.6%, the color fastness was grade 4–5, and the anti-wrinkle performance was 27% higher than that of only-dyeing-fabric. Lou et al. [45] prepared a multi-aldehyde derivative (oxyRa) via partial oxidation of raffinose with sodium periodate, and developed a novel “anti-wrinkle first, then dyeing” finishing mode to balance anti-wrinkle properties and dyeing performance. OxyRa cross-linked with hydroxyl groups of cotton fibers through its aldehyde groups, while its residual hydroxyl groups supplemented the hydrophilic sites of the fibers. The optimal process obtained via RSM was as follows: catalyst concentration of 20.12 g/L, pH 4.32, and curing at 150 °C for 120 s. Under these conditions, the fabric exhibited a CRA of 245.23°, representing a 106.87% improvement over untreated fabric. TSR was achieved at 71.13%, WI at 68.11. Compared to DMDHEU, GA, and BTCA, fabrics treated with oxyRa using the “two-step process” exhibited a K/S value of 13.88 and a Δ*E* value of 3.49, demonstrating color characteristics closer to those of pure-dyed fabrics (K/S = 19.63). The dyeability ranking was oxyRa > BTCA > DMDHEU > GA. Asim et al. [46] addressed the problem that reactive printing and anti-wrinkle finishing of traditional cotton fabrics cannot be achieved simultaneously by adopting a 2^2^·4^1^ mixed factorial design to systematically investigate the effects of shade percentage, anti-wrinkle agent concentration (Arkofix NEC), and fixation conditions on fabric color yield (K/S) and DCRA, and developed an integrated “padding-drying-printing-fixation” process. Analysis via Design Expert 7.0 showed that the three factors, as well as the interactions between shade percentage and fixation conditions, and between anti-wrinkle agent concentration and fixation conditions, were all significant (*p* < 0.05). The optimal process was the Econtrol urea-free fixation method (130 °C) combined with 3% shade percentage and 200 g/L anti-wrinkle agent, under which the K/S value reached 6.0 and the DCRA reached 129°, significantly superior to the saturated steam (102 °C/8 min) and hot air (150 °C/5 min) fixation methods. In this process, reactive dyes and anti-wrinkle agents were synergistically fixed via covalent bonds; although increasing the anti-wrinkle agent concentration slightly reduced the K/S value, it significantly improved the DCRA, achieving simultaneous compliance with requirements for full printing color and anti-wrinkle performance. The model R^2^ values were 0.9983 (for K/S) and 0.9904 (for DCRA), indicating good agreement between predicted and experimental values. Khasanova et al. [47] proposed a composite finishing system based on carboxyl-containing polymethylacrylate (PMA) latex and acetone–formaldehyde (ACF) resin, which balanced the improvement of anti-wrinkle properties and color fastness. Within this system, PMA latex formed a network film on the fiber surface and bonded to cellulose via ester linkages, whilst the ACF resin provided cross-linking support. Concurrently, DMEU and disodium ethylenediaminetetraacetate cross-linking agents were incorporated to optimize the finishing effect. Under optimized conditions, the treated fabric exhibited a CRA of 115° (a 30.7% increase compared with untreated fabric at 88°), soap washing color fastness of grade 5/5/4, and perspiration color fastness of grade 5/4/3; when the concentration of PMA-latex 1 was 200 g/L, the CRA reached a maximum of 190°, the fabric weight gain was 6.5%, and the wash resistance remained at 1.67% after eight washing cycles. This system simultaneously improves fabric abrasion resistance and color brightness (ΔV_c_ = 2001 kd/m^2^), and the fabric treated with PMA-latex 1 has lower stiffness (626.6 mkH·cm^2^) and better hand feel, providing a new path for the integrated anti-wrinkle and color-fixing finishing of cotton fabrics. Guan et al. [48] addressed the problems of poor anti-wrinkle properties of silk after inkjet printing, high costs of traditional stepwise finishing, and adverse effects on dyeing results by developing an integrated “pretreatment-printing-steaming” process. A bifunctional epoxy resin (ethylene glycol diglycidyl ether) was incorporated into the pretreatment paste as a formaldehyde-free anti-wrinkle finishing agent to simultaneously achieve printing and anti-wrinkle functions. During steaming at 100 °C for 30 min, the epoxy resin reacted with silk cystine to form a cross-linked network, while reactive dyes combined with fiber hydroxyl groups to complete fixation. These two reaction processes proceeded without interfering with one another. Optimal process was determined as 12% o.w.f. epoxy resin, under which the fabric exhibited a DCRA of 241° and a WCRA of 255° (increases of 19.3% and 50% compared with untreated fabric, respectively). The K/S value was comparable to that of traditional sodium alginate pretreatment, with soap washing color fastness of grade 3–4 and light fastness of grade 4–5; the tensile strength loss was only 14.2%, significantly lower than 23.3% at a concentration of 16% epoxy resin. This process is straightforward to operate, significantly enhanced the WCRA of silk while ensuring the color quality and fastness of printed fabrics. It effectively resolves the longstanding issue in traditional methods where pretreatment adversely affects printing, and post-treatment damages color fastness.

### 2.4. Multi-Functional Anti-Wrinkle Finishing Agent

Textile functional finishing is a core technical means to enhance the added value of textiles and expand their application scenarios [49,50]. However, most of the current functional finishing processes are carried out as independent operations, which not only increase the steps of production process and raise processing costs but also severely limit production efficiency and industrialization potential. Therefore, if anti-wrinkle finishing agents are used as the core carrier and endowed with multiple functional features through molecular modification technologies (e.g., chemical modification by introducing flame-retardant functional groups, physical doping of functional nanoparticles, etc.), it is expected to realize the simultaneous implementation of multiple functional finishing processes including anti-wrinkle, flame-retardant, antibacterial, and anti-ultraviolet finishing. Tu et al. [51] synthesized a nitrogen-containing heterocyclic compound with ammonium phosphate ester groups (PNH). PNH could be used as a cross-linking agent to form P-O-C covalent cross-linking bonds with hydroxyl groups of cotton fibers and then improve anti-wrinkle properties. Meanwhile, good flame retardancy also could be achieved via P-N synergistic effect. Under optimized conditions, the treated fabric exhibited a limiting oxygen index (LOI) of 42.7%, a CRA of 234.8°, and a peak heat release rate (pHRR) of 86.1%, lower than that of untreated fabric. Xie et al. [52] synthesized a DOPO-based ammonium phosphonate polymer (SH) via a one-pot method and proposed a “kill three birds with one stone” formaldehyde-free finishing strategy to simultaneously endow cotton fabrics with durable flame-retardant, anti-wrinkle, and anti-ultraviolet functions. SH could form P-O-C covalent cross-links with cellulose through its numerous ammonium phosphonic groups, thereby enhancing the anti-wrinkle performance; meanwhile, the phosphobenzophenyl groups in DOPO possessed both flame-retardant and ultraviolet absorption properties. Under optimal process conditions, the fabric treated with 15 wt% SH exhibited a CRA of 258.1°, while the fabric treated with 25 wt% SH had a LOI of 41.1% and an ultraviolet protection factor (UPF) of 285.4.

**Figure 2:**
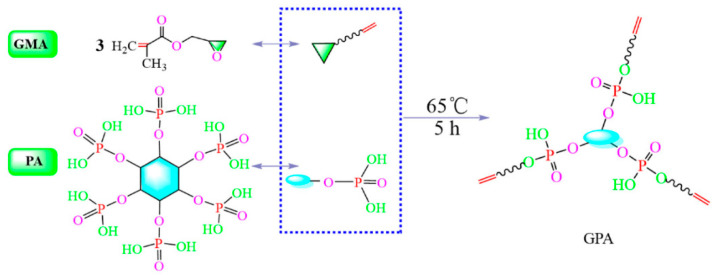
Reaction mechanism of PA and GMA [53] (Bingying Cheng et al., Materials, 2022, 16(1): 286).

**Figure 3:**
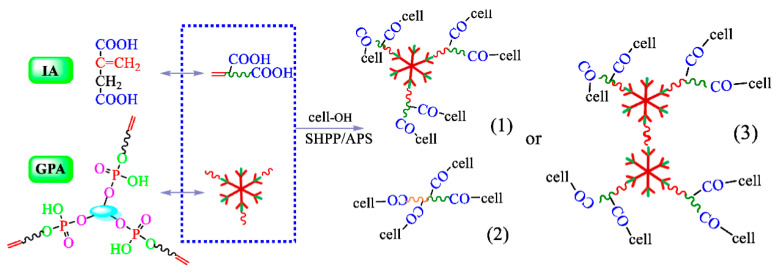
Graft reaction principle of cotton fabrics [53] (Bingying Cheng et al., Materials, 2022, 16(1): 286).

Cheng et al. [53] prepared an anti-wrinkle finishing agent integrated flame-retardant property through esterification reaction between phytic acid (PA) and glycidyl methacrylate (GMA), followed by in situ copolymerization with itaconic acid (IA) (Figure 2 and Figure 3). In this molecular design, vinyl phytic acid (GPA) provided phosphorus to endow fabric flame retardancy, while IA cross-linked with hydroxyl groups of cotton fibers to improve anti-wrinkle properties. Under optimized conditions, treated fabric exhibited a LOI of 28.6%, a CRA of 265°, and a residual carbon yield of 48% at 700 °C (only 10% for untreated fabric). Khoddami et al. [54] proposed synchronizing the antibacterial finishing of silver nanoparticles (AgNPs) with the conventional wrinkle-resistant finishing (cross-linking agent Fixapret ECO, film-forming resin Cellofix ME) of cotton fabrics, thereby developing a multi-functional finishing process that combines antibacterial properties, wrinkle resistance, and excellent durability. The study used pure cotton plain fabrics as a blank sample; the differences in finishing performance between two finishing methods, the pad-dry method with anti-wrinkle finishing agents alone, and the pad-dry-cure method with synergistic use of silver nanoparticles and anti-wrinkle finishing agents, were compared. Results showed that the antibacterial effect was positively correlated with the concentration of silver nanoparticles and dependent on the bacterial cell wall structure. The optimal concentration was 100–150 ppm for Gram-positive bacteria (Staphylococcus aureus), while for Gram-negative bacteria (*Escherichia coli*, *Pseudomonas aeruginosa*), the optimal concentration was 300 ppm due to the lipopolysaccharide layer on the outer cell wall hindering particle penetration. The addition of silver nanoparticles had no significant negative impact on the fabric’s CRA.

Chitosan is a natural polymer derived from the deacetylation product of chitin and is commonly used in the anti-wrinkle finishing of fiber fabrics such as cotton, linen, and silk [55]. Its mechanism of action mainly involves forming cross-linked structures between fiber molecules to enhance the elastic recovery ability of fibers, thereby reducing the generation of wrinkles. Meanwhile, the amino groups in chitosan molecules are protonated in an acidic environment, combining with proteins or nucleic acids in bacterial cells to interfere with enzyme activity and metabolic processes, thereby inhibiting bacterial growth and exhibiting certain antibacterial properties [56]. Samanta et al. [57] developed an environmentally friendly finishing solution that combined anti-wrinkle and antibacterial properties while preserving the mechanical properties and whiteness of jute fabrics. Using CA as a cross-linking agent, it covalently bonded simultaneously with jute cellulose and chitosan to construct a three-dimensional jute fiber–CA–chitosan cross-linked network, thereby enhancing the fabric’s wrinkle resistance. Simultaneously, the amino groups of chitosan formed ionic bonds with the carboxyl groups of CA, further enhancing antimicrobial and anti-mold properties. The cross-linked structure enhanced the fixation of the finishing agent on the fiber surface, achieving simultaneous improvement in wrinkle resistance and mold-resistance alongside enhanced wash durability. Under the optimal process parameters, 10% CA + 1.0% chitosan + 6% NaH_2_PO_2_, cured at 150 °C for 5 min, the DCRA reached 244° (a 62.7% increase compared with untreated fabric at 150°); the TSR rate after 21 days of soil burial was 81%, significantly higher than that of single CA treatment (50%) and single chitosan treatment (29.5%), showing a significant synergistic effect. In terms of wash resistance, after 5 washing cycles, the DCRA remained at 238°, and the strength retention rate was 72% after soil burial, showing slight performance degradation and good durability. Rouhani Shirvan et al. [58] developed an eco-friendly finishing scheme with both anti-wrinkle and antibacterial properties. This system utilized natural and biocompatible chitosan as a core material and selected CA and BTCA as cross-linking agents. Among them, chitosan formed a three-dimensional network within the fabric through esterification cross-linking with fiber hydroxyl groups or via ionic attraction between amino and carboxyl groups, simultaneously endowing fabric wrinkle resistance and antibacterial properties. When using the system comprising 7% (*w*/*v*) CA + 0.8% (*w*/*v*) chitosan + NaH_2_PO_2_, the treated cotton fabric exhibited a 100% antibacterial rate against Staphylococcus aureus and a wash fastness rating of 3.5–4. In addition, by using hydroxypropyl chitosan nanoparticles and BTCA combined treatment on tussah silk, the anti-wrinkle performance of the silk can be significantly improved; combining low-molecular-weight chitosan with DMDHEU to treat cotton fabric can endow the fabric with washable anti-wrinkle properties. Huang et al. [59] developed an integrated finishing agent combining pupal shell chitosan with CA and NaH_2_PO_2_, which endowed treated fabric synergetic antibacterial and wrinkle-resistant properties. Under optimal conditions, the DCRA of this fabric reached 242°, while the wet WCRA reached 186°, representing improvements of 29.4% and 34.8%, respectively, compared to untreated fabrics. Patankar et al. [60] developed a simple method for synthesizing nitrogen–phosphorus-rich green multi-functional chemical agents for fire-retardant, UV-protective, and antibacterial cotton fabrics. Chitosan was obtained from chitin via deacetylation. Extracted chitosan was then chemically modified with melamine and sodium pyrophosphate as nitrogen and phosphorous sources, respectively, and glyoxal as a cross-linking agent. The treated cotton fabrics presented excellent fire-retardant, UV-protective, and antibacterial properties. The LOI and UPF for the highest concentration treatment were found to be more than 30 and 100, respectively, whereas there was nearly 99% reduction in the bacterial colonies on treated cotton. In terms of mechanical properties, with the loading amount of modified chitosan increased, the treated cotton fabric gradually became harder, resulting in an increase in its bending length and consequent decrease in CRA. When the highest concentration of modified chitosan was applied, the total CRA of treated fabric was 116°. Ibrahim et al. [61] selected three types of nanoparticles (ZrO-NPs, ZnO-NPs, and TiO_2_-NPs) and incorporated them separately into five traditional finishing formulations, namely easy-care/softener, anti-ultraviolet, water-and-oil repellent, antibacterial, and flame-retardant formulations, and adopted the pad-dry-cure process to treat three types of fabrics, including 100% pure cotton, cotton/polyester (65/35), and cotton/polyester (50/50). The effects of different MO-NPs (ZrO, ZnO, and TiO_2_) and fabric types on the multi-functional finishing performance were systematically compared. Results indicated that the cotton/polyester (50/50) fabric treated with TiO_2_-NPs-modified easy-care formulation achieved a CRA of 276°, representing a 41.5% increase compared with untreated fabric (195°). The cotton/polyester (50/50) fabric treated with TiO_2_-NPs-modified anti-ultraviolet formulation reached an UPF of 130, far exceeding that of untreated fabric (20). ZnO-NPs modified antibacterial formulation exhibited the optimal antibacterial effect, with inhibition zone diameters of 24.5 mm against Staphylococcus aureus and 23.0 mm against *Escherichia coli*. The pure cotton fabric treated with ZnO-NPs modified flame-retardant formulation had its combustion time extended from 4 s to 22 s, showing a significant improvement in flame-retardant properties. The cotton/polyester (50/50) fabric treated with TiO_2_-NPs-modified water-and-oil repellent formulation achieved a water repellency rating of 90% and an oil repellency rating of Grade 8, with a UPF of 112. Wu et al. [62] synthesized a hydroxyl silane coupling agent with multiple siloxane branches (KHS), which was then reacted with polyurethane prepolymer and linear hydroxyl silicone oil (PMX500) to prepare a silicon-modified polyurethane finishing agent (KHS-PU), achieving the simultaneous improvement of fabric smoothness, elasticity, and anti-wrinkle properties. The polyurethane segments in KHS-PU provided film-forming property and elasticity, while the siloxane segments endowed smoothness. Under optimized conditions, the fabric surface friction coefficient decreased to 0.17 (a 23% reduction compared with untreated fabric of 0.22), and the CRA increased by more than 25°. Schramm et al. [63] adopted the sol–gel method, using (3-glycidoxypropyl) trimethoxysilane (GPTMS) as the organosilane and metal alkoxides (aluminum isopropoxide (AIP), titanium tetraisopropoxide (TTP), zirconium tetrabutoxide (ZTB)) to develop a formaldehyde-free anti-wrinkle finishing system for cotton fabrics. The epoxy groups in GPTMS underwent ring-opening to form diols, which then cross-linked with the hydroxyl groups on cotton fibers, thus enhancing the anti-wrinkle performance. Under optimized conditions, the fabric’s DCRA reached 289° (51.3% increase compared with untreated fabric of 191°). Hydrophobic components such as methyltriethoxysilane can be incorporated into this system, resulting in a contact angle of 154.0°; pretreatment with BTCA followed by GPTMS finishing can further increase the DCRA to 299°. Schramm et al. [64] synthesized an organic–inorganic hybrid finishing agent composed of (3-triethoxysilylpropyl) succinic anhydride (TESP-SA) and melamine (MEL) via the sol–gel method, which was applied to the anti-wrinkle modification of cotton fabrics. The carboxyl groups generated by the hydrolysis of TESP-SA reacted with the amino groups of melamine to form polyamic acid, which was converted into imide groups during high-temperature curing and cross-linked with the hydroxyl groups of cotton fibers. Under optimized conditions, the DCRA of treated fabric was increased by 60.3% compared with the untreated fabric.

## 3. Conclusions and Prospect

Anti-wrinkle finishing of textiles is a key technology to address the core pain point of fabric wrinkling tendency, and, in recent years, remarkable achievements have been made in the fields of performance synergy, eco-friendliness, process integration, and multi-functional integration. As for performance synergy, dynamic balance between anti-wrinkle properties and mechanical performance, hydrophilicity, and yellowing resistance has been achieved through the molecular design of flexible crosslinkers and precise regulation of composite systems. To achieve ecological sustainability, through relying on natural product modification, organic–inorganic hybridization, and biosynthesis technologies, the large-scale application of formaldehyde-free eco-friendly finishing agents has been promoted. In terms of processes and functions, the limitations of traditional independent finishing have been broken, and the simultaneous realization of anti-wrinkle properties and other functions such as dyeing, printing, flame retardancy, and antibacterial properties has been accomplished. In the future, textile anti-wrinkle finishing will continue to focus on greenization (e.g., development of bio-based materials), high performance, and multi-functional synergy, deepen the optimization of molecular structure, process parameters, and physical property regulation, comprehensively improve the anti-wrinkle efficiency, environmental attributes, and comprehensive application value of textiles, and provide core support for the green and sustainable development of the textile industry.

## Figures and Tables

**Table 1:** Structure and performance of traditional and novel eco-friendly finishing agents.

Structure	Performance	Example for Comparison
Carboxylated polyaldehyde sucrose [12]	CRA 258°, WI 72.1, TSR > 65%	Comparable to that of DMDHEU and GA
Isocyanate-terminated polysiloxane [13]	DCRA 302.6°, WCRA 229°, TSR 99%	-
Polycarboxyl-terminated trichlorotriazine derivatives [14,15]	DCRA 328.3°, WCRA 211.3°, TSR 98.3%	BTCA: DCRA 328.7°, WCRA 169.7°, TSR 88%
MA + NaH_2_PO_2_ [16]	CRA 275°, warp/weft/tear strength retention rate: 52%, 46%, 57%	DMDHEU: CRA 274°
DMDHEU + WPU + PEW-g-MAH [17]	Wrinkle recovery rate 83.71%, TSR 82%	DMDHEU/WPU: Wrinkle recovery rate 77.27% DMDHEU: TSR 59%
Modified DMDHEU [18]	CRA 263°, warp TSR rate 77.03%	CA: CRA 223°, warp TSR rate 74.92%
Itaconic acid [19]	CRA 158.2°	Slightly inferior to that of BTCA and MA
Tetra-aldehyde oxidized sucrose [20]	CRA 260°	-
BTCA + APTES [21]	DCRA 243.6°, TSR 78.5%, WI 17.1	-
Silicon-containing epoxy [22]	DCRA 267°, WCRA 275°	-

## Data Availability

No new data were created or analyzed in this study. Data sharing is not applicable to this article.
